# The modified G8 screening tool to predict post-operative complications and survival after robot-assisted radical cystectomy – a pilot study

**DOI:** 10.1186/s12894-026-02111-7

**Published:** 2026-03-17

**Authors:** Zein Alhamdani, Fabian Obrecht, Kapil Sethi, Damien Bolton, Gabriel Froelicher, Christoph Schregel, Hubert John, Beat Foerster

**Affiliations:** 1 Department of Surgery, University of Melbourne, Austin Health, Melbourne, VIC Australia; 2 Department of Urology, Kantonsspital Winterthur, Brauerstrasse15, Winterthur, 8401 Switzerland

**Keywords:** Radical cystectomy, Robot-assisted surgery, Modified G8 score, Frailty, Bladder cancer, Postoperative complications, Urinary diversion, Geriatric assessment

## Abstract

**Objective:**

To evaluate whether the modified G8 (mG8) screening tool predicts severe postoperative complications and 90-day mortality in patients undergoing robot-assisted radical cystectomy (RARC).

**Methods:**

We performed a single centre retrospective cohort study of patients who underwent RARC with ileal conduit or orthotopic neobladder diversion between 2011 and 2020 at a tertiary centre. Preoperative frailty was assessed using the mG8 score. Severe complications were defined as Clavien–Dindo grade ≥ 3. Univariable and multivariable logistic regression analyses were performed to evaluate the association of the mG8 score with severe complications and 90-day mortality.

**Results:**

A total of 155 patients were included. The median age was 69 years, and 49 patients (31.6%) had an impaired mG8 score (≥ 6). Severe complications occurred in 67 patients (43.2%), and 90-day mortality occurred in 10 patients (6.5%). In univariable analysis, an impaired mG8 score was significantly associated with severe complications (OR 2.94, 95% CI 1.46–5.93, *p* = 0.002). After adjusting for age group, the mG8 score remained an independent predictor (OR 2.65, 95% CI 1.29–5.45, *p* = 0.008). An impaired mG8 score was also associated with increased 90-day mortality (OR 5.72, 95% CI 1.46–22.81, *p* = 0.015). Age ≥ 76 years predicted higher mortality in univariable analysis but was not associated with severe complications in adjusted models.

**Conclusion:**

The mG8 screening tool is a promising predictor of major postoperative complications and 90-day mortality following RARC. Frailty, rather than chronological age or comorbidity burden, appears to better identify high-risk patients. Prospective multicentre studies are warranted to validate the role of the mG8 score in preoperative risk stratification for radical cystectomy.

**Supplementary Information:**

The online version contains supplementary material available at 10.1186/s12894-026-02111-7.

## Introduction

Bladder cancer is one of the most common malignancies globally [1]. Given the aging population, urologists globally are being faced with the dilemma of patients presenting at a later age with advanced malignancy where cystectomy would offer patients a curative treatment for their disease given its higher success rate [2, 3]. However, despite the oncological outcomes and a minimal invasive approach, robot-assisted cystectomy, especially in elderly patients, has a high risk of major complications [4–7]. Thus, this decision typically involves careful consideration of urologists in selecting the correct patient cohort to offer surgical treatment to, and then counselling patients about the risks to make a shared decision. As a result, clinicians have created screening tools to help discriminate between fit older patients who are likely to tolerate standard treatment and vulnerable patients who would benefit from alternative treatments tailored to their health [8].

One of the most widely known indices used in clinical decision-making is the Charlson Comorbidity Index (CCI) [9]. While primarily designed to predict long-term mortality (10-year survival), it has been used in clinical practice to assess a patient’s overall risk for adverse events following surgery [10–12]. However, its focus on long-term outcomes rather than short-term postoperative complications limits its utility in surgical contexts where short- and medium-term risks need to be considered.

The American Society of Anesthesiologists (ASA) score is commonly used to classify high-risk patients undergoing major surgery. However, studies have shown that in elderly patients undergoing radical cystectomy, the ASA score may not reliably predict the rates of major complications [13].

In contrast, the modified G8 (mG8) screening tool, developed by Martinez-Tapia et al., is a modified version of the G8 screening tool and has shown promise in predicting overall outcomes in cancer patients [14, 15]. This tool has demonstrated the ability to assess both frailty and comorbidities, making it a valuable predictor of short- and long-term survival at 1 and 3 years, in addition to determining which patients may need further assessment prior to forming a decision. However, it has not been used to predict surgical outcomes. The G8 screening tool has shown to be an effective tool at predicting post-operative outcomes in major urological surgeries performed for malignancy when compared to age or CCI [16]. The authors have found that a G8 score under 14 predicts higher rates of complications, and a score of under 10 makes a patient extremely high risk for complications [16, 17].

The modified G8 score is a shorter and easier to use screening tool based off the G8 screening tool that can be used more readily in daily clinical practice.

This is a pilot study to investigate whether the shorter and easier to use mG8 screening tool can forecast severe post-operative complications and survival following RARC to help guide the decision-making process for urologists.

## Methods

### Study design and population

We conducted a single-centre retrospective cohort study of patients who underwent robot-assisted radical cystectomy (RARC) with either intracorporeal or extracorporeal urinary diversion between 2011 and 2020 at our tertiary centre. Urinary diversions included ileal conduit and Studer’s orthotopic neobladder. Patients were included if they had complete data on preoperative modified G8 (mG8) screening with exception of the self-assessment item, and postoperative outcomes.

### Baseline characteristics and geriatric screening tools

**Fig. 1:**
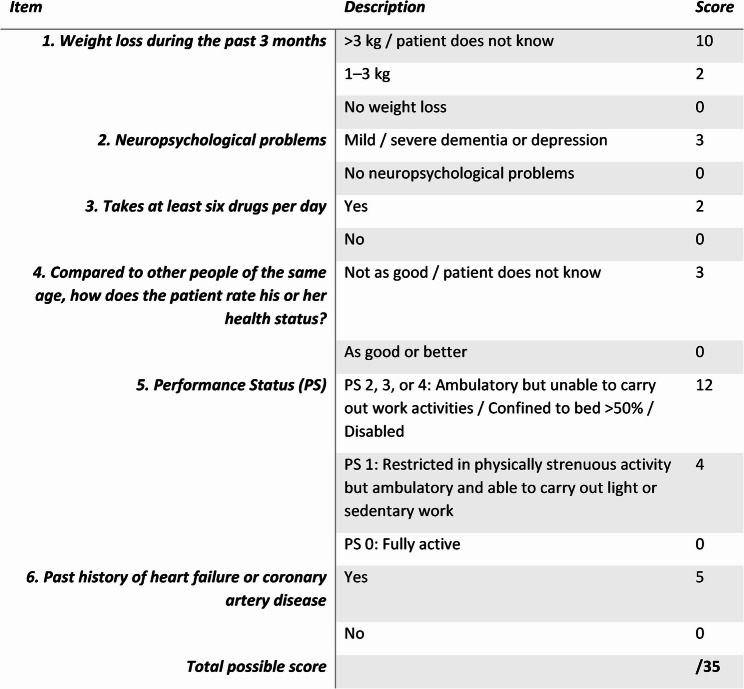
The modified G8 screening tool as published by Martinez-Tapia et al

Baseline characteristic assessed included age, body-mass index, sex, whether they had received neoadjuvant treatment and urinary diversion type. Furthermore, the mG8 score [15] was used to assess frailty preoperatively. The mG8 consists of six items that assess factors such as weight loss in the last 3 months, neuropsychological problems, polypharmacy, patient beliefs of own health status compared to others, performance status and past history of heart failure or coronary artery disease (Fig. 1). Body weight loss was assessed within three weeks prior to surgery during the preoperative anaesthetic assessment. The self-assessment item was considered zero in absence of the respective data. The individual mG8 components were obtained from pre-operative assessments; however, the composite mG8 score was calculated retrospectively for this pilot study, as the self-assessment component was not routinely collected and a uniform scoring approach was applied across the cohort. An impaired mG8 score was defined as a score of ≥ 6, in line with previous studies using this threshold to identify frail patients [14]. Additionally, the CCI was evaluated for each patient. Age was categorised into < 65, 66–74 and ≥ 75 years to reflect clinically relevant age groups rather than a binary definition of geriatric status. In oncogeriatric screening studies enrolling patients aged ≥ 65 years, the prevalence of geriatric abnormalities and their association with adverse outcomes increases progressively with age. Age stratification was therefore incorporated to assess whether the modified G8 screening tool predicted outcomes independently of chronological age [17, 18].

### Outcome measures

We focused on 90-day postoperative complications and 90-day mortality as primary outcomes. Complications were classified according to the Clavien-Dindo classification, with Grade ≥ 3 considered severe complications. 90-day mortality was defined as all-cause death occurring within 90 days following surgery. Peri- and postoperative data was collected from our clinical information system.

### Statistical analysis

Descriptive statistics were used to summarize patient characteristics, with continuous variables reported as median (interquartile range [IQR]) and categorical variables as frequencies (%). We used logistic regression to assess the association between different possible predictors and the occurrence of severe postoperative complications (Clavien-Dindo Grade ≥ 3) and mortality. Univariable analysis was performed to evaluate the association between impaired mG8 score and 90-day postoperative complications and mortality, as well as other factors such as age, Charlson Comorbidity Index (CCI), and age groups. Multivariable logistic regression was used to adjust for potential confounders to evaluate whether the mG8 score remained independently associated with severe complications and to compare the mG8 score to other predictive tools. Odds ratios (ORs) with corresponding p-values were calculated for each analysis. A p-value < 0.05 was considered statistically significant. We calculated the receiver operating characteristic–area under the curve (ROC-AUC) to evaluate the performance accuracy. The clinical net benefit of the model was assessed by decision curve analysis, which can be interpreted as a comparison between the use of modified G8 score, giving the percentage of patients saved from severe complications after RARC within a certain threshold probability. Internal validation was performed using bootstrap resampling (500 iterations) to assess model calibration.

All statistical analyses were performed using STATA Version 16.0 (StataCorp LLC. College Station, TX, USA).) Given the retrospective design and limited number of outcome events, a parsimonious multivariable modelling approach was used to minimise overfitting; variables were selected based on univariable association, with age group retained a priori due to its clinical relevance.

### Ethical considerations

The cantonal Review Board of Zurich approved the study protocol (BASEC-No. 2017 − 01260). We elaborated this observational study according to the STROBE statement for cohort studies. All patient data were de-identified to ensure confidentiality.

## Results

### Patient characteristics

A total of 155 patients underwent robot-assisted radical cystectomy (RARC) with intracorporeal or extracorporeal urinary diversion between 2011 and 2020. The median age was 69 years (IQR 63–76), and 118 patients (76.1%) were male. Ileal conduit diversion was performed in 113 patients (72.9%). Baseline clinical characteristics are summarised in Table 1.

**Table 1:** Perioperative data

Variable	Results
Number of patients, n	155
Age, years	69 (64–76)
Sex, n (%)	
Male	118/155 (76.1%)
Female	37/155 (23.9%)
BMI, kg/m^2	26 [23.1–28.4]
Urinary diversion type, n (%)	
Ileal conduit	113 (72.9%)
Neobladder	42 (27.1%)
Pathological Tumor stage	
(y)pT0	8 (5.2%)
(y)pTa/Tis/T1	48 (31.0%)
(y)pT2	34 (21.9%)
(y)pT3/T4	48 (31.0%)
Other Origin (Prostate, Cervix, Endometrium)	9 (5.8%)
Functional	8 (5.2%)
Postoperative complications, n (%)	
Clavien-Dindo Grade 3 or higher	67/155 (43.2%)
Clavien-Dindo Grade 5	10/155 (6.5%)
Charlson comorbitity Index, n (%)	
0–2	134/155 (86.5%)
≥ 3	21/155 (13.5%)
Impaired modified G8 score (≥ 6 points), n (%)	49/155 (31.6%)

Results are given as median [IQR] or number (percentage), as appropriate

An impaired modified G8 (mG8) score (≥ 6) was present in 49 patients (31.6%), and a Charlson Comorbidity Index (CCI) ≥ 3 was observed in 21 patients (13.5%). The self-assessment component of the mG8 score was unavailable for all patients; 17 of 155 patients had baseline mG8 scores between 3 and 5, for whom inclusion of this item could have resulted in reclassification above the ≥ 6-point threshold. The detailed distribution of modified G8 scores across the cohort is provided in Supplementary Table 1.

### Postoperative outcomes

Severe postoperative complications (Clavien–Dindo grade ≥ 3) occurred in 67 patients (43.2%). Ten patients (6.5%) died within 90 days of surgery. A detailed breakdown of the complication is depicted in the Supplementary Table 2.

### Predictors of 90-day severe complications

On univariable analysis, an impaired mG8 score was associated with an increased risk of severe postoperative complications (OR 2.94, 95% CI 1.46–5.93; *p* = 0.002) (Table 2). Age category, sex, body mass index, and comorbidity burden were not significantly associated with severe complications. Similarly, surgical technique of urinary diversion (intracorporeal versus extracorporeal) was not associated with severe 90-day postoperative complications (OR 0.93, 95% CI 0.46–1.88; *p* = 0.85). Patients aged ≥ 76 years demonstrated a non significant trend towards higher risk (OR 2.15, 95% CI 0.93–4.98; *p* = 0.07).

**Table 2:** Uni- and multivariable analysis predicting severe 90-day complications (Clavien-Dindo Grade 3 or higher)

Univariable model			Multivariable model	
	OR (95% CI)	*p*-value	OR (95% CI)	*p*-value
Age	1.03 (0.99–1.06)	0.14		
Sex				
Male	Ref.			
Female	1.33 (0.64–2.80)	0.4		
BMI	0.96 (0.88–1.05)	0.4		
Urinary diversion type				
Ileal conduit	Ref.			
Neobladder	0.57 (0.27–1.19)	0.13		
Surgical technique of urinary diversion				
extracorporeal	Ref.			
intracorporeal	0.93 (0.46–1.88)	0.85		
Charlson comorbidity Index				
0–2	Ref.			
≥ 3	1.53 (0.61–3.85)	0.4		
Age group				
≤ 65 years	Ref.		Ref.	
66–75 years	1.11 (0.52–2.37)	0.8	1.09 (0.50–2.37)	0.8
≥ 76 years	2.15 (0.93–4.98)	0.07	1.69 (0.71–4.06)	0.2
Impaired modified G8 score	2.94 (1.46–5.93)	0.002	2.65 (1.29–5.45)	0.008

*BMI* Body mass index

On multivariable analysis, impaired mG8 remained independently associated with severe complications (OR 2.65, 95% CI 1.29–5.45; *p* = 0.008). Chronological age was not independently associated with severe complications, including patients aged ≥ 76 years (OR 1.69, 95% CI 0.71–4.06; *p* = 0.2) or those aged 66–75 years (OR 1.09, 95% CI 0.50–2.37; *p* = 0.8). Using the predefined cutoff of mG8 ≥ 6, the score demonstrated a sensitivity of 44.8% and specificity of 78.4% for predicting Clavien–Dindo grade ≥ 3 complications (Table 3). The positive predictive value was 61.2% and the negative predictive value was 65.1%.

**Table 3:** Association between modified G8 score and major postoperative complications

Modified G8 score	No major complication	Major complication	Total
< 6 point	69	37	106
≥ 6 points	19	30	49
Total	88	67	155

ROC analysis demonstrated modest discrimination of the mG8 score for predicting major complications, with an AUC of 0.62 (Supplementary Fig. 1). Decision curve analysis showed a clinical net benefit of 0.07 using a basic model with age groups and 0.13 adding the modified G8 score to the basic model at the threshold probability of 40% (Supplementary Fig. 2). Bootstrapped calibration of the model demonstrated good agreement between predicted and observed outcomes, with a calibration slope of 0.99 and a calibration intercept close to zero (− 1.17 × 10⁻⁸). Internal validation using 500 bootstrap resamples showed stable estimates, with bootstrap standard errors of 0.34 for the calibration slope and 0.18 for the intercept.

### Predictors of 90-day mortality

On univariable analysis, impaired mG8 was strongly associated with 90-day mortality, conferring a 5.7-fold increased risk (OR 5.72, 95% CI 1.46–22.81; *p* = 0.015) (Table 4). Advanced age (≥ 76 years) was also associated with increased mortality (OR 10.3, 95% CI 1.22–87.76; *p* = 0.033). Comorbidity burden (CCI ≥ 3) was not significantly associated with 90-day mortality (OR 3.03, 95% CI 0.71–13.06; *p* = 0.13).

**Table 4:** Univariate analysis predicting 90-day mortality

Univariable model		
	OR (95% CI)	*p*-value
Age	1.012(1.02–1.23)	0.013
Sex		
Male	Ref.	
Female	1.40 (0.34–5.71)	0.6
BMI	0.96 (0.79–1.17)	0.7
Urinary diversion type		
Ileal conduit	Ref.	
Neobladder	∞ (not calculable)	> 0.001
Charlson comorbidity Index		
0–2	Ref.	
≥ 3	3.02 (0.72–12.75)	0.13
Age group		
≤ 65 years	Ref.	
66–75 years	1.64 (0.14–18.61)	0.7
≥ 76 years	10.29 (1.21–87.50)	0.033
Impaired modified G8 score	5.72 (1.41–23.19)	0.015

*BMI* Body mass index

No deaths occurred among patients undergoing neobladder reconstruction; therefore, an odds ratio could not be calculated for this subgroup. Multivariable analysis for 90-day mortality was not performed due to the limited number of events.

## Discussion

In this retrospective cohort study of 155 patients undergoing robot-assisted radical cystectomy (RARC), we found that an impaired modified G8 (mG8) score was significantly associated with severe postoperative complications and 90-day mortality. In multivariable analysis, the mG8 score remained independently associated with severe complications, while age and Charlson Comorbidity Index (CCI) did not. Furthermore, an impaired mG8 score was a strong predictor of 90-day mortality, with a particularly high risk observed in patients aged 76 years or older.

The findings of this study suggest that the mG8 score may be a more reliable predictor of postoperative outcomes in elderly patients undergoing RARC than traditional measures such as age and CCI. This supports previous research suggesting that frailty, rather than chronological age alone, is a key determinant of surgical risk and postoperative morbidity in this patient population [19–21].

Our findings demonstrate promising results in the utility of the mG8 score in predicting adverse outcomes in cancer patients. We found that age ≥ 76 years was not independently associated with severe complications or mortality after adjusting for frailty and comorbidity indices. It is noteworthy that none of the patients in the neobladder group experienced 90 day mortality. This finding likely reflects the stringent local criteria for neobladder selection, whereby only relatively healthy patients are offered this diversion. Despite this inherent selection of lower risk patients, urinary diversion type was not associated with differences in 90 day complication rates.

While other frailty predictors currently exist, the current literature demonstrates that there are no reliable frailty indices that have been shown to predict outcomes post radical cystectomy for bladder cancer. For instance, some studies have shown that a modified frailty index (mFI) might predict major complications post radical cystectomy [22, 23]. However, these studies were limited by their sample size and heterogeneity in study protocol. In addition, Woldu et al. assessed major complications post-operatively utilising the American Society of Anaesthesiologists classification (ASA), CCI and mFI [24]. In their cohort of 346 patients, none of these classification systems were able to predict complications.

Another study compared a simplified frailty index (sFI)to the ASA and found that a score of 3 + out of 5 had much better predictive value at major complications within 30 days of surgery when compared to ASA [25]. However, out of 5516, only 123 patients were classified in the higher risk category in their sFI scoring system. As a result, while it shows promise, it’s applicability is limited. While it can help exclude very high-risk patients, it does not sufficiently differentiate between those at intermediate risk which limits its ability to fully guide clinical decision making. In contrast, prior oncogeriatric studies have demonstrated improved prognostic discrimination when screening scores are analysed in graded risk categories rather than using a binary threshold alone [14]. Accordingly, future studies may consider stratifying modified G8 scores into low (0–5), intermediate (6–13) and high (≥ 14) risk groups.

The G8 has been shown to be useful in predicting postoperative complications in older patients undergoing major uro-oncological surgery, with scores ≤ 14 associated with higher complication rates and graded categorisation further improving risk discrimination [16]. However, several domains of the standard G8 are primarily nutrition-focused or rely on subjective self-assessment, reflecting its original role as a general geriatric screening tool rather than a surgical risk instrument. Consequently, not all components may be equally relevant to short term perioperative outcomes. The modified G8 was developed to address these limitations by simplifying and refocusing assessment on domains more closely related to physiological reserve and perioperative vulnerability, which may make it more applicable in the uro-oncological surgical setting.

Given its ability to predict both postoperative complications and mortality, the mG8 score may be a valuable tool for urologists when assessing elderly patients prior to RARC. It may help identify patients at higher perioperative risk who could benefit from preoperative optimisation strategies, such as nutritional support, physical therapy, or formal geriatric assessment. From a practical perspective, the mG8 score could be readily integrated into routine preoperative workflows as a rapid screening tool to support multidisciplinary decision-making and shared discussions regarding surgical risk, alternative treatment pathways, and alignment of treatment choices with patient goals and physiological reserve, rather than serving as an exclusion criterion [26].

This study has several limitations. First, it is a retrospective cohort study, which may introduce selection bias and limit the generalizability of the results. Moreover, given the retrospective nature of this pilot study, data for the self assessment item of the mG8 tool were not available. As this item contributes up to 3 points, assigning a value of zero when missing may have led to an underestimation of frailty in some patients. This could have resulted in misclassification of frail individuals into the non frail category, which would tend to weaken the observed association between the mG8 score and postoperative outcomes. In this cohort, only 17 of 155 patients had baseline mG8 scores between 3 and 5, for whom inclusion of the self assessment item could have resulted in reclassification above the ≥ 6-point threshold. Despite this potential underestimation of frailty, we still found a strong relationship between impaired mG8 scores and both major complications and 90 day mortality. Because the self assessment item represents only a small proportion of the overall score, the number of patients whose classification would have meaningfully changed is therefore limited. Although detailed data on ERAS implementation and institutional learning-curve effects were unavailable, we performed an additional analysis accounting for surgical technique of urinary diversion. The absence of an association between intracorporeal and extracorporeal diversion and severe postoperative complications suggests that variation in surgical technique alone did not drive the observed outcomes, although residual temporal heterogeneity remains possible Complications occurring beyond 90 days were not analysed, and therefore the relationship between the modified G8 score and longer-term postoperative complications was not explored. However, this approach is consistent with existing literature, which predominantly focuses on operative and 90-day outcomes to assess short-term postoperative risk, as these outcomes are most relevant to perioperative surgical decision-making. Prospective collection of full mG8 data is planned at our institution to better understand the impact of the self reported health component on the performance of the tool. The data on frailty and postoperative outcomes were collected from a single institution, which may not fully represent the broader population of patients undergoing RARC. Additionally, while the mG8 score was associated with complications and mortality, other unmeasured factors such as psychological status or social support might also play a role in these outcomes. Finally, the relatively small sample size limits the power of some of the statistical analyses, and prospective validation in a larger cohort is necessary.

Future studies should validate the predictive value of the mG8 score in larger, multicenter cohorts to confirm its utility in broader populations. Prospective research is also needed to evaluate whether interventions targeting frailty, such as prehabilitation or early postoperative care, can reduce the risks associated with high mG8 scores. Furthermore, comparative studies between the mG8 score and other frailty indices, such as the modified frailty index (mFI) or the Clinical Frailty Scale (CFS), could provide valuable insights into the most effective tools for preoperative risk stratification.

## Conclusion

This is the first study to investigate the role of mG8 in predicting post-operative outcomes post RARC. An impaired mG8 score has been shown to be a promising predictor of major complications post robot-assisted radical cystectomy. Larger prospective multi-centre trials comparing outcomes of this frailty index compared to others are required to validate its role in pre-operative risk stratification.

## Supplementary Information

Supplementary Material 1.

Supplementary Material 2.

Supplementary Material 3.

Supplementary Material 4.

## Data Availability

The datasets generated and/or analysed during the current study are not publicly available due to institutional and patient privacy restrictions but are available from the corresponding author on reasonable request.
