# Supplementary material for: The modified G8 screening tool to predict post-operative complications and survival after robot-assisted radical cystectomy – a pilot study
Source: BMC Urol. 2026 Mar 17;26:104. doi: 10.1186/s12894-026-02111-7 (PMC13107592; doi:10.1186/s12894-026-02111-7)

Supplementary Figure 1: Area under the ROC curve for the proposed multivariable logistic regression model predicting major complications in 155 patients who underwent robot-assisted radical cystectomy. ROC: receiver operator characteristics. The multivariable model includes modified G8 score.


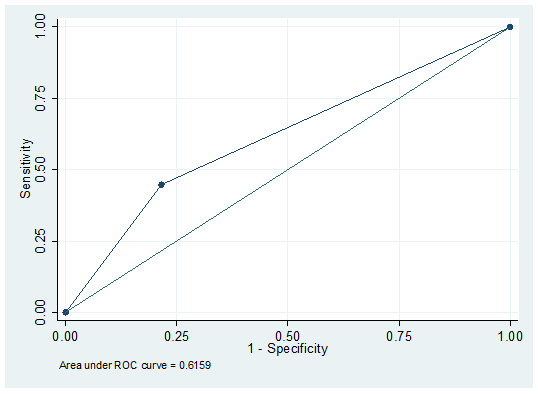

Supplement: Supplementary file 1 — Supplementary Material 1. [file 12894_2026_2111_MOESM1_ESM.docx]
