# Supplementary material for: The modified G8 screening tool to predict post-operative complications and survival after robot-assisted radical cystectomy – a pilot study
Source: BMC Urol. 2026 Mar 17;26:104. doi: 10.1186/s12894-026-02111-7 (PMC13107592; doi:10.1186/s12894-026-02111-7)
Supplement: Supplementary file 2 — Supplementary Material 2. [file 12894_2026_2111_MOESM2_ESM.docx]

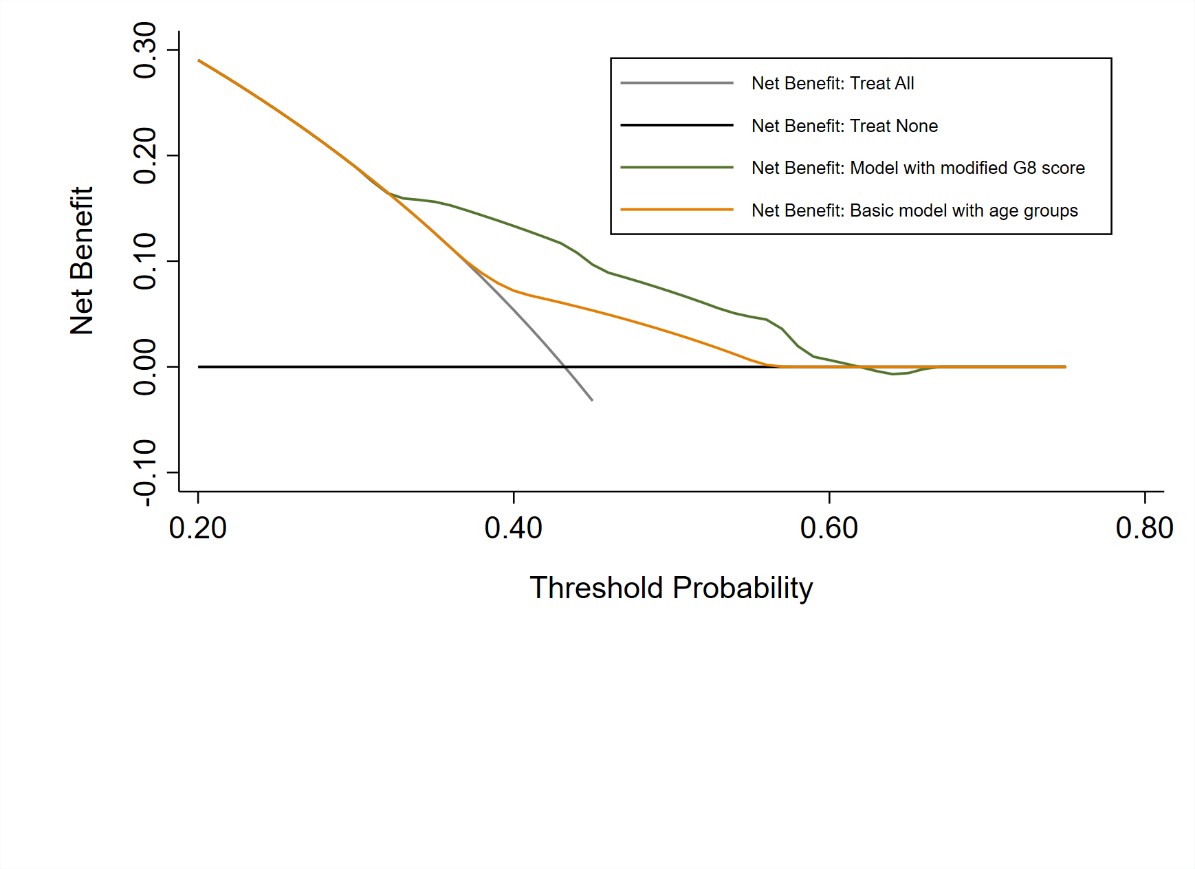


Supplementary Figure 2: Decision curve analysis demonstrating the clinical net benefit of the advanced model incorporating modified G8 score compared to the basic model with age groups only. Clinical net benefit has a ready clinical interpretation. At a threshold probability of 40% clinical net benefit values of 0.07 and 0.13 can be interpreted as follows: “Compared to using the basic model with age groups, performing robot-assisted radical cystectomy on the basis of the model with modified G8 score is the equivalent of a strategy that found 6 per hundred patients saved from major postoperative complications”
