# Supplementary material for: The modified G8 screening tool to predict post-operative complications and survival after robot-assisted radical cystectomy – a pilot study
Source: BMC Urol. 2026 Mar 17;26:104. doi: 10.1186/s12894-026-02111-7 (PMC13107592; doi:10.1186/s12894-026-02111-7)
Supplement: Supplementary file 3 — Supplementary Material 3. [file 12894_2026_2111_MOESM3_ESM.docx]

**Supplementary Table 1**: Detailed Distribution of the modified G8 score

| **Modified G8 Score (points)** | **Number of Patients (%)** |
| --- | --- |
| **0** | 72 (46.5%) |
| **1 – 5** | 34 (21.9%) |
| **6 – 10** | 34 (21.9%) |
| **11 – 15** | 10 (6.5%) |
| **16 - 21** | 5 (3.2%) |
