# Supplementary material for: The modified G8 screening tool to predict post-operative complications and survival after robot-assisted radical cystectomy – a pilot study
Source: BMC Urol. 2026 Mar 17;26:104. doi: 10.1186/s12894-026-02111-7 (PMC13107592; doi:10.1186/s12894-026-02111-7)
Supplement: Supplementary file 4 — Supplementary Material 4. [file 12894_2026_2111_MOESM4_ESM.docx]

**Supplementary Table 2:** Detailed description of the major complication rate (Clavien Dindo grade 3 or higher).

| **Clavien-Dindo complication Grade** | **Number of Patients (%)** |
| --- | --- |
| **IIIa** | 13 (8.4%) |
| **IIIb** | 34 (21.9%) |
| **IVa** | 8 (5.2%) |
| **IVb** | 2 (1.3%) |
| **V** | 10 (6.5%) |
